# Investigating the Characteristics of Nano-Graphite Composites Additively Manufactured Using Stereolithography

**DOI:** 10.3390/polym16081021

**Published:** 2024-04-09

**Authors:** Ariyana Dwiputra Nugraha, Vishnu Vijay Kumar, Jessie Puteri Gautama, Ardi Wiranata, Kevin Gausultan Hadith Mangunkusumo, Muhammad Ibnu Rasyid, Rachmat Dzanzani, Muhammad Akhsin Muflikhun

**Affiliations:** 1 PLN Research Institute, Jakarta 12760, Indonesia; 2 Mechanical and Industrial Engineering Department, Gadjah Mada University, Yogyakarta 55284, Indonesia; vishnu.vijay@u.nus.edu (V.V.K.); jessieputerigautama@mail.ugm.ac.id (J.P.G.); ardi.wiranata@ugm.ac.id (A.W.);; 3 International Institute of Aerospace and Engineering & Management, JAIN (Deemed-to-be-University), JGI Global Campus, Bangalore 562112, India

**Keywords:** stereolithography, additive manufacturing, nano-graphite, composite

## Abstract

Stereolithography has emerged as a recent method in fabricating complex structures with high accuracy. Components using resin have poorer properties. The current study investigates the improvement in the properties of nano-graphite composites fabricated by the SLA technique. The properties are compared for plain resin and 0.2%, 0.5%, 1%, 3%, and 5% (*w*/*v*) of nano-graphite mixed with the UV-curable resin. Various analyses were conducted, including viscosity, UV spectroscopy, moisture content, water absorption, gel content, tensile, bending, hardness testing, and microscopic characterization. The results from the experiments showed a difference in the results of each percentage of the specimen tested, such as the specimen property, which shows that the greater the percentage of nano-graphite added (5%), the opaquer the specimen will appear and less light will be reflected. Viscosity testing shows that the greater the percentage of nano-graphite added to the resin, the greater the viscosity. UV spectroscopy testing produced information about the electronic structure and the structure of molecules, such as their composition, purity, and concentration. Observations from the moisture content analysis found that the moisture content in specimens with higher percentages of nano-graphite affected physical and mechanical properties, leading to easier warping, cracking, decreased strength, etc. Tensile and bending testing shows that the greater the percentage of nano-graphite added, the greater the effect on physical and mechanical properties, including fracture. However, certain tests did not consistently yield significant variations among specimens when different percentages of nano-graphite were added, as particularly evident in chemical resistance testing. This study offers valuable insights into the application of nano-graphite composites fabricated via the SLA method.

## 1. Introduction

Graphite is a form of carbon that is widely used in various industries due to its high thermal stability, chemical resistance, and electrical conductivity [1]. Some typical applications of graphite include use in lubricants, battery electrodes, the partition of insulator walls, refractory crucibles, nuclear reactors as moderators, heat exchangers, and thermal-ballistic protection in spacecraft and electric vehicles [2,3,4]. Carbon-based materials are a well-accepted reinforcement in additive manufacturing of composites [5,6]. However, graphite is not commonly used as a material for additive manufacturing. The traditional method of producing materials with graphite involves powder metallurgy. The binder, when heated, softens and allows the uniform reinforcement of graphite powder with homogeneous adhesion. The inability of such methods to produce complex parts with high precision and high porosity, in addition to the high cost involved and environmental concerns related to use, demanded a more economical and flexible method of production. Most of the existing methods employ subtractive manufacturing methodologies but their limitation in producing complex shapes gave rise to the growth of additive manufacturing techniques like selective laser sintering (SLS) [7], stereolithography (SLA) [8,9,10], laminated object manufacturing (LOM) and binder jet printing (BJP) [11,12]. Fused deposition modelling (FDM) is a widely used 3D printing method, but the products sometimes suffer from delamination, poor accuracy, and heat shrinking of the resin [11,13]. SLA has emerged as an efficient method for fabricating products with excellent accuracy and complex structures [14,15].

The UV resin used in SLA usually results in poorer mechanical properties. Studies were conducted to improve the mechanical properties and add functionalities. CNT [16], cellulose nanocrystals [17], and graphene [18] were used to improve the functionalities of the resin, but the resultant product showed poor mechanical properties [19,20,21]. Several studies investigated the improvement in the properties of powder reinforced in 3D printers, finding an improvement in the mechanical properties for the composite method [22,23]. Another study on graphene-based 3D structures showed improvements in electrical conductivity and mechanical properties [24,25]. Polymer-based bonding of graphite has emerged as a cost-effective method to produce complex geometries. A recent study on a novel method of additively manufacturing denser graphite structures by the binder jetting method was investigated. In the study, the authors integrated binder jet printing (BJP) and  cold isostatic pressing (CIP) to produce graphitic parts with a high density and less porosity. Controlling the density of graphite powder is crucial in determining the properties of the composite. A lower density results in post-processing issues, with reduced strength and lower wear resistance. Although many studies have focused on improving resin properties [26,27,28], there is significant untapped research potential in exploring nano-graphite fillers through the stereolithography (SLA) technique.

The current study explores the improvement in the properties of nano-graphite composites through SLA with varying filler concentrations. Various analyses were conducted, including viscosity, UV spectroscopy, moisture content, water absorption, gel content, tensile, bending, hardness testing, and microscopic characterization. The properties when using different concentrations of nano-graphite were compared to identify the optimal filler proportion. This study offers valuable insights into the analysis of nano-graphite composite fabrication using SLA.

## 2. Materials and Methods

The materials used for additive manufacturing were C-PRO WELD nano-graphite powder from Yogyakarta, Indonesia and ANYCUBIC (wavelength 405 nm) UV-sensitive resin from ANYCUBIC Company, US. The properties of graphite powder and resin are given in Table 1 and Table 2, respectively.

### 2.1. Fabrication of the Specimens

The specimens are fabricated using the SLA additive manufacturing technique. Photon Mono SE from ANYCUBIC from Indonesia (properties are given in Table 3) was employed for additive manufacturing of the UV-curable resin and the nano-graphitic (NG) mixture. A total of 6 types of specimens were fabricated: plain UV resin; 0.2%, 0.5%, 1%, 3% and 5% (*w*/*v*) of NG powders mixed in the UV-curable resin. The reason for using 0 to 5% is that the optimum concentration is unknown and a trial-and-error technique was employed to investigate the optimum percentage for enhanced properties.

The resin and NG powders are mixed at different proportions using a THINKY planetary centrifugal bubble-free mixer ARE-310 from Japan, where mixing was performed for 20 min and defoaming for 20 min to obtain a homogeneous suspension.

The suspension of varying graphitic concentrations was fed into the additive manufacturing machine and printed as per the specifications given in Table 3. After printing, all specimens were washed in an isopropyl alcohol bath for 20 min, followed by UV curing by direct UV exposure for 20 min for each specimen to ensure complete curing.

### 2.2. Viscosity Analysis

The viscosity of the resin and nano-graphitic mixture was measured using an NDJ-8S digital rotary viscometer at UGM, Indonesia using rotor configuration #1, with a uniform speed of 30 rpm at 25 °C. A comparative study was conducted using pure UV resin, and 0.2%, 0.5%, 1%, 3%, and 5% NG were incorporated into the resin.

### 2.3. UV Spectroscopy

The individual absorption and transmittance of the resin and nano-graphitic mixtures were computed using a Thermo Scientific GENESYS™ 50 UV-Visible Light Spectrophotometer at UGM, Indonesia. The measurement was performed at a wavelength of 190 nm to 1100 nm with a 5 nm division.

### 2.4. Moisture Content

The moisture content in 0.2%, 0.5%, 1%, 3%, and 5% nano-graphitic composites was computed using an MB-95 OHAUS moisture analyzer at UGM, Indonesia. The specimen was heated at 125 °C for 10 min to measure the percentage of moisture content in each sample. The weight difference before and after heating was also estimated, including the initial weight (W_initial_) and final weight (W_final_) of the specimens. The moisture content (%) was estimated as given in Equation (1).

(1) $$\mathrm{Moisture}\;\mathrm{content}\;\left(\%\right)=\frac{\left(W_{\mathrm{final}}-{\;W}_{\mathrm{initial}}\right)}{W_{\mathrm{initial}}}\times 100$$

### 2.5. Water Absorption

The water absorption was evaluated as per the ASTM D-570 standard [29] for the 0.2%, 0.5%, 1%, 3% and 5% specimens. Five specimens of each type were first dried in the oven to obtain a constant weight reading and dipped in water for 24 h. The initial weight (W_initial_) and the final weight (W_final_) were measured. The water absorption percentage was calculated as per Equation (2).

(2) $$\mathrm{Water}\;\mathrm{absorption}\;\left(\%\right)=\frac{\left(W_{\mathrm{final}}-{\;W}_{\mathrm{initial}}\right)}{W_{\mathrm{initial}}}\times 100$$

### 2.6. Gel Content

The gel content in the UV-cured graphitic composite samples was measured by estimating the weight difference before and after curing. The gel content (%) was calculated using Equation (3), where W_bc_ is the weight of the specimen before curing and W_ac_ is the weight of the specimen after curing.

(3) $$\mathrm{Gel}\;\mathrm{content}\;\left(\%\right)=\frac{\left(W_{\mathrm{bc}}-{\;W}_{\mathrm{ac}}\right)}{W_{\mathrm{bc}}}\times 100$$

### 2.7. Chemical Resistance

The chemical resistance of the UV-cured composite was estimated according to the ASTM D-543 standard [30]. The specimens of the 0.2%, 0.5%, 1%, 3%, and 5% composites were dipped separately in HCl (10% *w*/*w*) and NaOH (10% *w*/*w*) solution for 24 h at 25 °C. The composites were visually analyzed for any surface defects, deformation or dissolution of particles at the end of the test.

### 2.8. The Tensile Test

The tensile test was performed following ASTM D-683 type 4 standard [31] using a CRN-50 CARSON uniform tensile testing machine from Taiwan. The samples were fixed between the grips of a fixed and movable gauge, and a uniform tensile force was applied at 5 mm per min crosshead speed. Tabbing was provided to prevent grip area failure, and five specimens of each type were tested. The post-tensile tested specimens are studied, and the nature of failure is investigated. The properties like tensile strength, Young’s modulus, and strain at break were also computed. The microscopic analysis of post-tested specimens was also conducted.

### 2.9. The Bending Test

A three-point bending test was performed on the UV-cured composites as per the ASTM D-790 standard [32]. Five specimens of each type were tested at a moving crosshead speed of 2 mm per minute. The flexural properties of the graphitic composites were estimated and compared.

### 2.10. The Hardness Test

Two types of hardness testing were conducted to analyze the hardness of the nano-graphitic composite. Firstly, Shore hardness (D) was performed as per the ASTM D-2240 standard [33] on five different specimens of each type. Also, Leebs hardness testing was used in predicting the hardness values in the Leebs scale on five different specimens of each type.

### 2.11. Microstructural Characterization

Microstructural characterization of the specimens was conducted using a Dino-lite AM 4515T8 series digital microscope at UGM, Indonesia with a 1.3 MP sensor resolution, a 700–900× adjustable focus and magnification. The post-tensile tested samples are analyzed and recorded.

## 3. Results and Discussions

### 3.1. Specimen Properties

**Figure 1:**
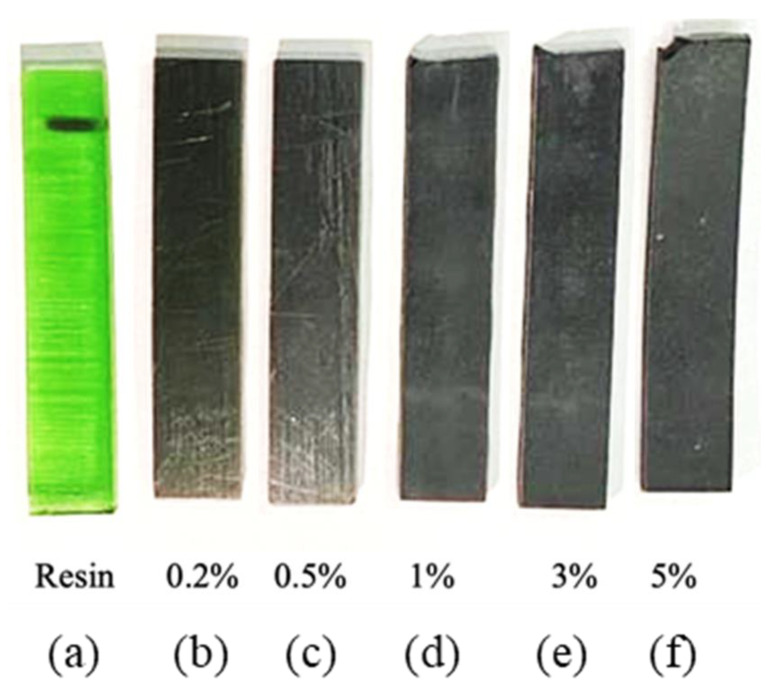
Fabricated specimens: (**a**) resin, (**b**) 0.2%, (**c**) 0.5%, (**d**) 1%, (**e**) 3%, and (**f**) 5%.

The fabricated specimens of six different types of resin, 0.2%, 0.5%, 1%, 3% and 5% composites, are shown in Figure 1. The pure resin was translucent, whereas those with NG reinforcement had varying optical natures. The 5% resin was mostly opaque, and the translucency decreased with a decreasing percentage of NG added to the resin. The differences in optical properties observed among the fabricated specimens highlight the impact of nano-graphite (NG) reinforcement on the resin. As the percentage of NG in the composites increases, the opacity of the specimens intensifies, with the 5% composite exhibiting predominantly opaque characteristics. Conversely, the translucency diminishes as the proportion of NG decreases. These variations in optical nature underscore the influence of NG concentration on the visual appearance of the composites, offering valuable insights into their structural composition and potential applications. The same appearance corroborates a study on graphite particulate in hybrid composite materials [34].

### 3.2. Viscosity Analysis

The viscosity measurement results are shown in Table 4. The measurement was taken for pure resin and the various NG resin mixtures. From the results, it can be inferred that the addition of NG to the control resin resulted in a reduction in solution viscosity. The greater the amount of NG powder mixed into the resin system, the more viscous the liquid resin. This result corroborates the findings in the literature—the addition of glass powder increased pure resin viscosity [13].

### 3.3. UV Spectroscopy

**Figure 2:**
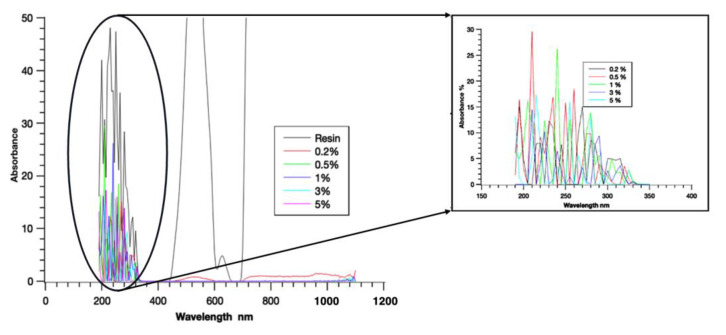
UV spectroscopy showing the absorbance of the specimens.

The results obtained from UV spectroscopy can provide information about the electronic structure and properties of molecules, such as their composition, purity, and concentration. The absorbance and transmittance are important factors that determine the properties of materials. The absorbance and transmittance spectra of resin and different NG composites are presented in Figure 2. From the figure, it can be inferred that the behavior of the pure resin and the NG-added resin were different. The absorbance spectra showed that the resin had zero absorbance close to the wavelength of 750 nm, but this was not the case for the NG composite. Similarly, for the transmittance spectra, the maximum transmission for pure resin occurred at 500 nm, whereas it was in the range of 200–300 nm for the NG composite. It was also interesting to note that the 0.2% NG showed a result similar to plain resin, but the absorbance values were higher, whereas in transmittance, the 0.2% NG showed non-zero transmittance in the range above 400 nm, with zero transmittance at 700 nm. The other proportions of NG show variations, and this study helps identify the level of absorbance and transmittance change that occurred in pure resin with NG addition.

### 3.4. Moisture Content

**Figure 3:**
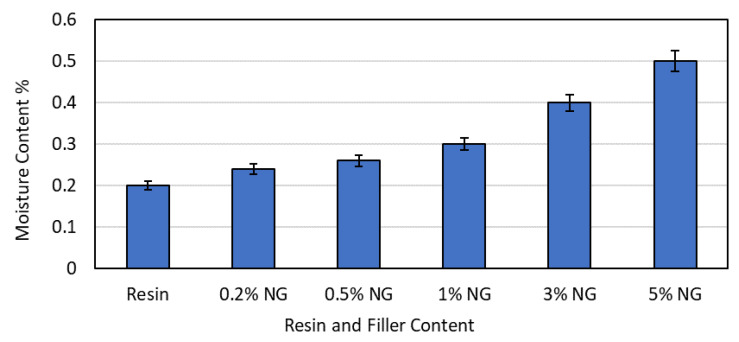
Moisture content observations for resin and NG composites.

The moisture content in a sample can have a significant impact on the physical and mechanical properties of the material and the final printed product. If the moisture content is too high, this can cause issues such as warping, cracking, and reduced strength in the printed parts [18]. This can also lead to poor surface end and adhesion problems between the layers of the printed material. Figure 3 shows the observed moisture content in the samples. From the result, it was observed that the moisture content increased with an increase in the NG percentage. The result corroborated a recent study using a nano-graphite mixture in additive manufacturing of multibinder geopolymer composites [19]. The 0.5% NG showed more water absorption as there may have been interfacial differences, resulting in pores through which the water molecules can easily penetrate. The overall results suggest that a greater addition of NG results in a composite with increased moisture content, which is undesirable.

### 3.5. Water Absorption

**Figure 4:**
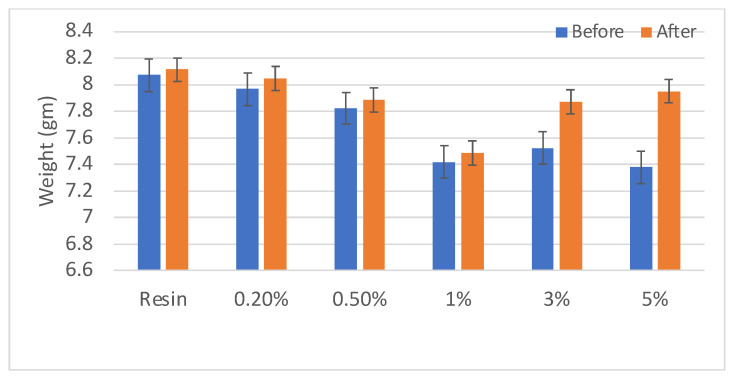
Water absorption properties of resin and NG composites.

From the results, it can be seen that the water absorption of the NG-reinforced composites increased with an increase in the amount of NG added. This was similar to the results for moisture content shown above, and the justification is similar. With a greater amount of NG, more voids are produced. This results in the water molecule being infused into those pores upon submerging. This increases moisture absorption and the weight of the composite post-experiment. This study was highly significant as increased water absorption results in a composite with reduced mechanical properties and dimensional characteristics. Surface treatments or coating may be provided to reduce the water absorption nature of 3D printed composites. The water absorption properties are presented in Figure 4. According to the data, the observed trend in the water absorption of the NG-reinforced composite corresponds to an increase in the amount of NG included. This trend matches the data for moisture content, demonstrating a continuous pattern. The explanation for this phenomenon is linked to the increased creation of voids with the addition of NG. These gaps act as conduits for water molecules to infiltrate during immersion, increasing moisture absorption and, as a result, increasing the weight of the composite after investigation.

### 3.6. Gel Content

The gel content in 3D printed structures shows the amount of polymer that has not undergone crosslinking during printing and post-curing. Lower gel contents resulted in a greater effect on mechanical properties and thermal stability. However, a high gel content percentage signifies more uncured resin and might result in brittleness or flexibility of printed parts.

**Figure 5:**
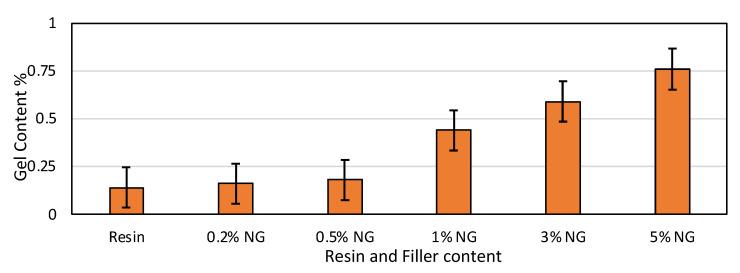
Gel content properties of resin and NG composites.

The gel contents of the resin and various NG composites are given in Figure 5. Five sets of similar specimens were used for each proportion to improve the reputability and reliability of the results. From the results, it can be inferred that the gel content percentage in the finished specimen increases with an increase in the proportion of NG addition. Compared with plain resin, the percentage increase in gel content for 0.2, 0.5, 1, 3 and 5% NG composites, respectively, is 14.2%, 28.5%, 21.4%, 32.1%, and 44.2%. It is interesting to note that the gel content increased nearly 4.5 fold when compared with plain resin and the 5% NG composite. The increase in gel content resulted in brittleness and a reduction in properties.

### 3.7. Chemical Resistance

The chemical resistance of resin and NG composites with HCl (acid) and NaOH (base) did not show any visible reactions. The surface of the materials was analyzed and no changes were recorded with the chemical treatments. These findings demonstrate the composite’s capacity to survive corrosive environments without deterioration or change, highlighting its applicability for applications that need resistance to acidic or alkaline conditions. To improve the chemical resistance of 3D printed parts under acid or base exposure, post-processing techniques like coating or annealing may be used.

### 3.8. The Tensile Test

**Figure A1:**
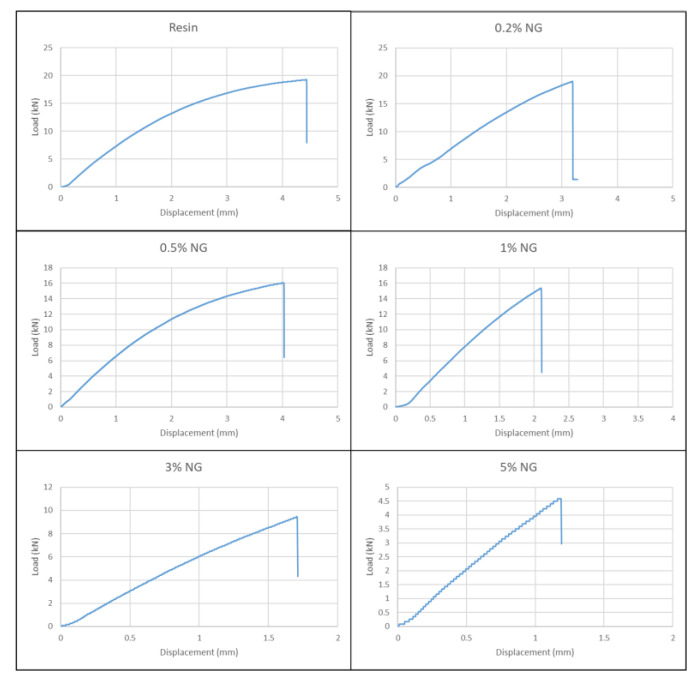
Tensile test results for resin and NG composites.

The tensile test results for resin and NG composites are given as load and displacement plots shown in Figure A1 in the appendix section. Five repetitive tests were conducted on each proportion, but one representation for each proportion is shown in the figure. The values are given tabulated as shown in Table 5. It was found that the greater the composition of the percentage added, the higher the influence on physical and material properties, as in the graphic above, it is shown that the greater the amount of nano-graphite, the higher the chance of specimen fracture. The fracture is caused by a high-stress concentration.

### 3.9. The Bending Test

**Figure 6:**
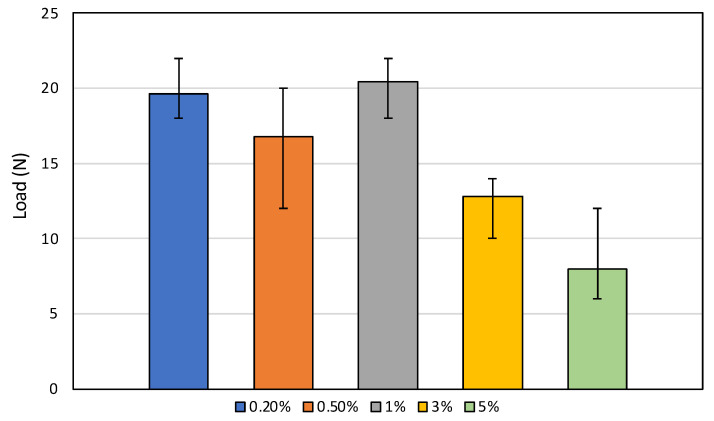
Maximum bending load test results for resin and NG composites.

The results from the bending testing for resin and NG composites are given in Figure 6. Five repetitive tests were conducted on each proportion and the results are shown in Figure 6. The bending load result shows differences between each filler percentage. While the 0.2% showed an average max load of 19.6 N, it decreased at 0.5%, with an average max load of 16.8 N. However, at 1% filler percentage, the load increased up to 20.4 N. It then decreased again at 3%, and 5% was the lowest with 12.8 N and 8 N, respectively. The filler affected the bending load by the interaction between the filler particle and the resin. Too much filler content increases crack initiation. 

The trend of the bending load decreasing initially at 0.5% NG filler and then increasing at 1% NG filler can be attributed to the interplay between the reinforcing effect of the nano-graphite particles and the agglomeration or dispersion state of the particles within the resin matrix. At a low filler content of 0.2%, the nano-graphite particles act as reinforcing agents, improving the load-bearing capacity of the composite compared to the neat resin. However, as the filler content increases to 0.5%, the particles may start to agglomerate or exhibit poor dispersion within the resin matrix. These agglomerates can act as stress concentration points, leading to a decrease in the overall mechanical properties, including the bending load. As the filler content further increases to 1%, the increased number of well-dispersed nano-graphite particles can effectively transfer the applied load from the resin matrix, resulting in an improvement in the bending load. This phenomenon is often observed in polymer composites, where an optimum filler content exists, beyond which the agglomeration effects become more prominent, leading to a decrease in properties.

### 3.10. The Hardness Test

**Figure 7:**
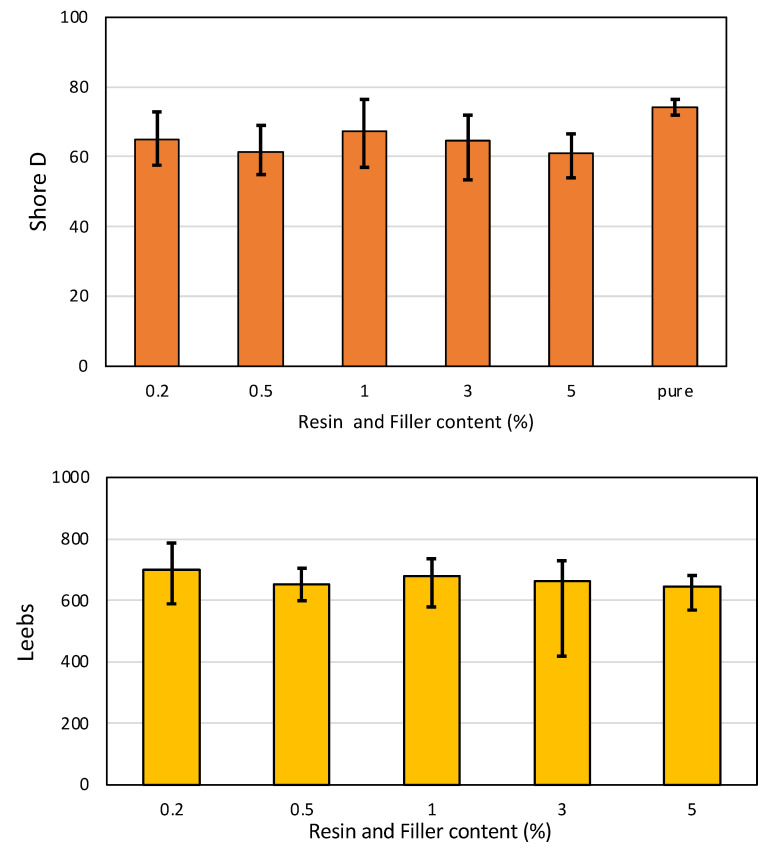
Hardness testing results (Shore D and Leebs) for resin and NG composites.

The results from hardness testing are depicted for resin and NG composites in Figure 7. Hardness was applied with Shore hardness and Leebs hardness. The results indicated that the difference between pure resin and powder addition was not crucial and had similar results based on the Shore D hardness. Further analysis using Leebs hardness for resin added with powder-based materials indicated that powder addition did not significantly affect the hardness value. This finding is that the graphite nanopowder can blend and disperse seamlessly into the resin system, thereby minimally impacting the hardness performance. The observation that the Shore D hardness of the nano-graphite (NG) composites remained consistent with the neat resin, despite the increased porosity and water absorption associated with higher NG loads, appears contradictory at first glance. However, this behavior can be explained by considering the factors influencing hardness and the potential mechanisms governing the water absorption process in these composites. Hardness, as measured by the Shore D test, is primarily influenced by the inherent properties of the resin matrix and interfacial adhesion between the filler and the matrix. The presence of well-dispersed and strongly bonded nano-graphite particles can reinforce the resin matrix, increasing its hardness. However, if agglomeration or poor interfacial adhesion occurs, the reinforcing effect may be diminished, leading to a decrease in hardness. On the other hand, water absorption in polymer composites is typically governed by the presence of voids, pores, or defects within the material. These voids can arise from various factors, such as incomplete curing, air entrapment during processing, or the introduction of filler particles that create interfacial gaps or voids within the resin matrix. In the case of the NG composites, a greater amount of nano-graphite particles may have led to the formation of more voids or defects within the resin matrix. These voids could be attributed to factors such as agglomeration of the NG particles, poor dispersion, or inadequate interfacial bonding between the NG and the resin. As a result, water molecules could penetrate and accumulate within these voids, leading to increased water absorption. However, despite the presence of these voids and increased water absorption, the hardness of the composites remained consistent with the neat resin. This observation suggests that the well-dispersed and strongly bonded NG particles within the resin matrix still contribute to the composite’s reinforcement and hardness, counteracting the voids’ potential negative effects on hardness.

### 3.11. Microstructural Characterization

**Figure 8:**
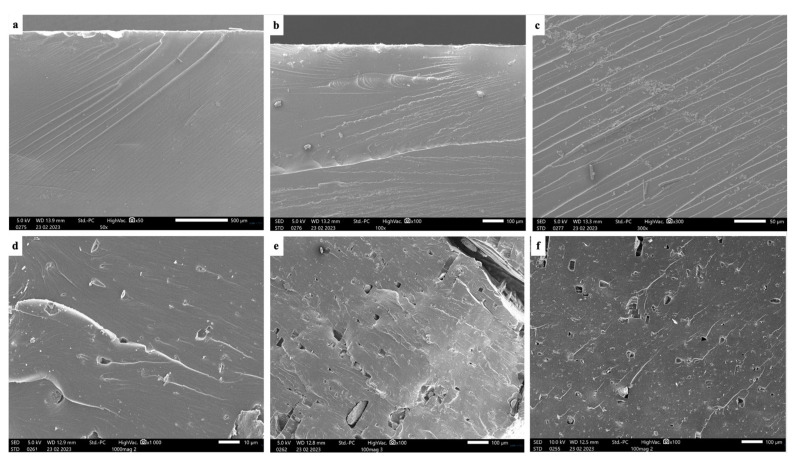
Microstructure of fracture surface: (**a**) pure resin, (**b**) 0.2% NG, (**c**) 0.5% NG, (**d**) 1% NG, (**e**) 3% NG, and (**f**) 5% NG.

The microstructural analysis uncovered significant insights into the fracture surfaces of various specimens, as illustrated in Figure 8. Observing the figure, it can be deduced that the fracture surface of the pure resin specimen, depicted in Figure 8a, appeared smooth, suggesting a ductile characteristic. 

As the percentage of NG increased to 0.2% and 0.5%, the number of fractal lines on the fractured surface increased, indicating a phenomenon of load transfer. With a further increase in the NG percentage, micropores began to form during both tensile and bending tests. A higher percentage of NG revealed deeper voids previously occupied by NG powders, suggesting that additional NG incorporation may lead to diminished properties and the formation of voids under load. Moreover, the fracture behavior at 5% NG shows a brittle failure nature compared to pure resin, showing a ductile nature. Additionally, as the NG concentration increased beyond 0.5%, the fractured surfaces exhibited a more pronounced presence of micropores and voids, particularly evident in specimens containing 3% and 5% NG. These voids appeared to be interconnected, suggesting potential pathways for crack propagation and structural weakness. The agglomeration of NG powders may also account for a reduction in property with the increased percentage of addition. Moreover, the formation of these voids seemed to coincide with regions where NG particles were densely packed, indicating a correlation between NG dispersion and void formation under mechanical loading conditions. This observation highlights the importance of optimizing NG concentration to balance material reinforcement with potential void formation and structural integrity.

Previous studies have explored the use of carbon-based fillers, such as carbon nanotubes (CNTs) and graphene, in polymer composites for AM, reporting improvements in mechanical properties, electrical conductivity, and thermal stability. However, the incorporation of NG in SLA-printed composites has not been extensively investigated, making this study a notable contribution to the field. The findings related to the viscosity analysis align with existing literature, which suggests that higher filler concentrations can lead to increased viscosity and potential challenges in printability [35]. However, the observed reduction in viscosity at 5% NG concentration is an interesting outcome that warrants further exploration. The observed changes in UV absorbance spectra due to the presence of NG are consistent with previous studies on carbon-based fillers [36], which have shown the ability to absorb and scatter UV radiation. This phenomenon can potentially affect the curing behavior and properties of the composite material, as highlighted in this study. The increased water absorption observed at 0.5% NG concentration is a critical finding, as it highlights the importance of optimizing filler dispersion and interfacial interactions to minimize the formation of pores and defects that can facilitate moisture ingress. Similar observations have been reported in other studies involving carbon-based fillers in polymer composites [37]. The significant increase in gel content at higher NG concentrations (5%) is an intriguing result, as it suggests potential brittleness and property reduction, which aligns with the observed decrease in tensile and bending loads. This phenomenon has been observed in other polymer composite systems, where excessive filler content can lead to agglomeration, stress concentration, and premature failure [38].

The chemical resistance of the NG composites to acidic and basic environments is a positive finding, as it indicates potential applications in various industries where exposure to harsh chemicals is a concern [39]. The findings provide new insights into SLA-printed nano-graphite composites, serving as a significant reference for future studies and possible applications in additive manufacturing.

## 4. Conclusions

Various characterizations of additively manufactured nano-graphite composites using the SLA technique have been conducted. The percentage of nano-graphite ranged from 0.2% to 5% (*w*/*v*) mixed with curable UV resin. 

The key findings from the research can be summarized as follows:

o
Viscosity analysis revealed that higher concentrations of nano-graphite (5% NG) resulted in a lower viscosity (130 mPa.s) of the composite material, potentially improving printability.
o
UV spectroscopy showed differences in the absorbance and transmittance spectra between the resin and NG composites, indicating potential changes in light-curing behavior.
o
Moisture content tests indicated that the 0.5% NG composite exhibited higher water absorption due to interfacial differences and the presence of pores.
o
Gel content tests demonstrated a nearly 4.5-fold increase in gel content for the 5% NG composite compared to plain resin, suggesting potential brittleness and property reduction at higher filler concentrations.
o
Chemical resistance tests showed no visible reaction or surface changes when the resin and NG composites were exposed to HCl acid and NaOH base.
o
Tensile testing revealed that higher nano-graphite percentages reduced the maximum load, with the 5% NG composite exhibiting the lowest tensile load (4.05 kN), possibly due to stress concentration and faster fracture initiation.
o
Bending tests indicated that 1% NG composites could increase the bending load compared to the resin only (20.4 kN), while higher concentrations (3% and 5% NG) significantly reduced the bending load, likely due to excessive filler content increasing the chance of crack initiation.

Overall, the research provides valuable insights into the effects of nano-graphite concentration on the properties of additively manufactured composites, highlighting potential trade-offs and optimal compositions for specific applications.

## Figures and Tables

**Table 1:** Properties of Resin.

Property	Value
Hardness	79 D
Viscosity (25 °C)	552 mPa.s
Curing wavelength	405 nm
Liquid density	1.1
Solid density	1.184 g/cm^3^
Shrinkage	7.10%
Tensile strength	23.4 MPa
Curing time	6–10 s
Elongation at break	14.20%

**Table 2:** Properties of Graphite.

Property	Value
Mesh size	150
Shape	Flake Graphite powder
Min ash	1.50%
Size	0.1–0.5 nm
Carbon content	98%

**Table 3:** Additive manufacturing parameters.

Property	Unit	Value
Layer thickness	mm	0.05
Normal exposure time	s	2
Off time	s	0.5
Bottom exposure time	s	30
Bottom layers	No	6
Z lift distance	mm	6
Z lift speed	mm/s	4
Z retract speed	mm/s	6

**Table 4:** Viscosity measurements.

Material	Viscosity (mPa.s)	Torq (%)
Resin	138	70
0.2% NG	137	69
0.5% NG	136	68
1% NG	134	67
3% NG	132	66
5% NG	130	65

**Table 5:** The average values of tensile test results of resin and NG composites.

Specimen	Load (kN)	Average Max Displacement (mm)
Resin	16.81	2.7756
0.2%	16.70	3.3928
0.5%	14.61	3.7564
1%	13.44	1.897
3%	8.82	2.308
5%	4.05	1.6516

## Data Availability

Data will be available on request.
